# Current status and continuing medical education need for general practitioners in Tibet, China: a cross-sectional study

**DOI:** 10.1186/s12909-024-05143-5

**Published:** 2024-03-08

**Authors:** Sen Yang, Huaxin Zhao, Hanzhi Zhang, Junpeng Wang, Hua Jin, Kyle Stirling, Xuhua Ge, Le Ma, Zhen Pu, Xiaomin Niu, Dehua Yu

**Affiliations:** 1 Department of General Practice, Research Center for General Practice, Yangpu Hospital, School of Medicine, Tongji University, 450 Tengyue Road, Yangpu District, Shanghai, 200090 PR China; 2 Department of General Practice, Lazi County Health Service Center, Xigatse, Tibet, 858100 PR China; 3 Department of Oncology, Shanghai Tenth People’s Hospital, Tongji University School of Medicine, Shanghai, PR China; 4 Medical Administration Affiliationision, Yangpu Hospital, Tongji University School of Medicine, Shanghai, 200090 PR China; 5 Crisis Technologies Innovation Lab, Luddy School of Informatics, Computing and Engineering, Indiana University, Bloomington, IN 47408 USA; 6 Department of Shanghai Lung Cancer Center, Shanghai Chest Hospital, Shanghai Jiao Tong University School of Medicine, 241 Huaihai West Road, Shanghai, 200030 PR China; 7 Shanghai General Practice and Community Health Development Research Center, Shanghai, 200090 PR China

**Keywords:** General practitioner, Teaching needs, Continuing medical education, Primary Health Care

## Abstract

**Background:**

The Tibetan area is one of China’s minority regions with a shortage of general practice personnel, which requires further training and staffing. This research helps to understand the current condition and demand for general practitioner (GP) training in Tibetan areas and to provide a reference for promoting GP education and training.

**Methods:**

We conducted a cross-sectional survey using stratified sampling targeting 854 GPs in seven cities within the Tibetan Autonomous Region, utilizing an online questionnaire. Achieving a high response rate of 95.1%, 812 GPs provided invaluable insights. Our meticulously developed self-designed questionnaire, available in both Chinese and Tibetan versions, aimed to capture a wide array of data encompassing basic demographics, clinical skills, and specific training needs of GPs in the Tibetan areas. Prior to deployment, the questionnaire underwent rigorous development and refinement processes, including expert consultation and pilot testing, to ensure its content validity and reliability. In our analysis, we employed descriptive statistics to present the characteristics and current training needs of GPs in the Tibetan areas. Additionally, chi-square tests were utilized to examine discrepancies in training needs across various demographic groups, such as age, job positions, and educational backgrounds of the participating GPs.

**Results:**

The study was completed by 812 (812/854, 95.1%) GPs, of whom 62.4% (507/812) were female. The top three training needs were hypertension (81.4%, 661/812), pregnancy management (80.7%, 655/812), and treatment of related patient conditions and events (80.5%, 654/812). Further research shows that the training required by GPs of different ages in “puncturing, catheterization, and indwelling gastric tube use” (64.6% vs. 54.8%, *p* = 9.5 × 10^− 6^) varies statistically. GPs in various positions have different training needs in “community-based chronic disease prevention and management” (76.6% vs. 63.9%, *p* = 0.009). The training needs of GPs with different educational backgrounds in “debridement, suturing, and fracture fixation” (65.6% vs. 73.2%, *p* = 0.027) were also statistically significant.

**Conclusions:**

This study suggests the need for targeted continuing medical education activities and for updating training topics and content. Course developers must consider the needs of GPs, as well as the age, job positions, and educational backgrounds of GPs practicing in the Tibetan Plateau region.

**Trial registration:**

Not applicable.

**Supplementary Information:**

The online version contains supplementary material available at 10.1186/s12909-024-05143-5.

## Background

General practitioners (GPs) play a vital role in providing a wide range of primary health care (PHC) services, including preventive care, treatment, and referrals for common diseases, chronic disease management, and health management [1, 2]. In the context of Tibet, a unique province in China with distinct characteristics such as a high-altitude, low-oxygen geographic environment, limited economic development, and specific cultural and historical traditions, the number and training of GPs are insufficient to meet the region’s future healthcare needs [3–5]. The quantity and quality of GPs in Tibet continue to be lower than the minimum requirements of the Chinese government, and this situation will be expected to worsen by 2030 [6]. Given the challenges faced by primary healthcare institutions and GPs in Tibet, it is crucial to identify and address the specific training needs of GPs to enhance the overall quality of PHC in the region.

The existing literature has highlighted constraints such as limited participation in training due to practical course attendance and time constraints in Chaya County, Tibet [7, 8]. Other researchers have evaluated the training needs of GPs in different regions of China, comparing the knowledge and skill requirements across the eastern, central, and western areas [9, 10]. Furthermore, a review of GP training needs in Ireland highlighted common CME needs such as updated protocols, geriatrics, chronic disease treatment, and dermatology [11]. Additionally, multiple studies have delved into the training needs of GPs in areas such as evidence-based medicine, tuberculosis control and management, and the care of vulnerable populations [12–14]. Despite there have been descriptions and evaluations of GP training needs, the published literature on GP training needs is very preliminary and not specific enough to support wider adoption, and research on GP training needs in the entire Tibet Autonomous Region is limited.

In Tibet, the training of physicians comprises basic medical education, clinical practice, and specialized training [7]. Medical students undergo 3–5 years of basic medical education at universities, focusing on mastering fundamental medical knowledge and skills. Subsequently, they engage in 1–3 years of standardized residency training and internship. Following this, doctors pursue further specialization through programs such as master’s or doctoral studies. To address the need for primary medical services, Tibet has introduced a GP training program, transforming rural doctors into GPs after a brief transfer training period or offering free training for medical students under the Rural Order Orientation Program, emphasizing general practice in primary health-care institutions. Additionally, Continuing Medical Education (CME) plays a vital role in Tibet, with GPs gaining further training at larger hospitals and participating in various educational opportunities such as study classes, academic conferences, and online education [15].In spite of this, the opportunities and number of GPs in Tibet who can receive CME are still very limited, making it far more difficult to meet the needs of local PHC.

Therefore, this study aims to fill this knowledge gap by investigating the current situation of GPs and their CME needs, specifically, our research questions are as follows:

1. What is the structure of GPs in Tibet in terms of age, gender, job position, practicing categories, professional positions and degree?
2. Are there differences in CME needs among GPs of different age groups?
3. Are there differences in CME needs among GPs with different positions and education degrees?

In brief, this study delved into the training needs of GPs in the Tibet Autonomous Region, offering valuable insights for the future development of CME programs for GPs.

## Methods

### Survey sample and participants

A multicenter, cross-sectional, descriptive survey methodology was used for this study. From March to August 2022, the survey study was carried out. Based on the population density, economic conditions and distribution of PHC institutions in Tibet, a stratified sampling method was used to survey seven cities, including the Lhasa, Xigaze, Nyingchi, Qamdo, Shannan, Nagqu, and Ngari Prefectures. From March to August 2022, we submitted the questionnaire to GPs in the seven cities listed above. The inclusion criteria for participants in the study population were as follows: (1) those working at PHC facilities such as primary hospitals, community health service centers, and village clinics and (2) those able to read and understand the questions included in the questionnaire. The exclusion criteria were as follows: (1) non-GPs and (2) individuals who were unable to complete the web-based survey questionnaire.

### Survey questionnaire development

The employed questionnaire is based on the classic six-step framework for curriculum development proposed by Kern et al. [16] (problem identification; needs assessment of the learners; educational goals and objectives; instructional strategies; implementation, and evaluation and feedback) with reference to the World Organization of Family Doctors (WONCA) core competence model for GPs [17]. For further guidance, we invited 18 experts, including 5 general practice professionals, 4 public health experts, 4 managers of PHC institutions, and 5 GPs in Tibetan areas, to review and revise the content of the questionnaire. This expert review process aimed to ascertain that each item in the questionnaire accurately reflects the intended dimensions, particularly focusing on the unique context of CME for GPs in Tibet. To further refine the questionnaire and enhance its applicability, a pilot study was conducted with a carefully chosen group of GPs. The feedback obtained from this study was instrumental in the iterative process of fine-tuning the questionnaire. Finally, the questionnaire is comprised of multiple dimensions, encompassing comprehensive data. This includes basic demographic and professional information of GPs, educational background, as well as specific needs for CME. It covers 8 aspects, consisting of a total of 15 questions, as shown below (Additional file 1):

A) basic information about the GPs (8 questions),
B) clinical skill training needs (8 options),
C) community chronic disease management skill training needs (7 options),
D) common chronic disease training needs (13 options),
E) public health service skill training needs (6 options),
F) special population health management training needs (8 options),
G) training needs for infectious diseases and handling of public health emergencies (5 options),
H) rehabilitation technology training needs (5 options).

In addition to obtaining basic information, each question allowed for more than one response. Taking into account the diversity of languages, we meticulously translated the questionnaire into Tibetan. During this process, we employed back-translation techniques to ensure the accuracy of the language and the appropriateness of the cultural context. Ultimately, we produced the survey questionnaire in both Chinese and Tibetan versions. The internal consistency of the questionnaire was thoroughly evaluated using Cronbach’s alpha, resulting in a high reliability coefficient of α = 0.87. This indicates a strong degree of consistency in the responses across various items of the questionnaire, reinforcing the reliability of our tool for assessing the educational needs of GPs. To further validate the reliability of our questionnaire, a test-retest method was employed. A subgroup of participants (*n* = 30) completed the questionnaire twice with a two-week interval. The high correlation coefficient (*r* = 0.82) observed between the two sets of responses confirms the stability and reliability of the questionnaire over time.

### Data collection and quality control

In March 2022, the online version of the questionnaire was developed using the Sojump software, a popular and user-friendly online survey tool in China. This platform was chosen for its reliability, ease of use, and features that facilitate effective data collection and management. The questionnaires were sent to the leaders of primary health services in Tibet through online WeChat (Quick Response) QR codes and then distributed to the managers of PHC institutions and GPs in seven cities in Tibet. Participants completed the questionnaire at their convenience, with an average completion time of approximately five minutes. The recruitment strategy involved coordination by research group staff members, who facilitated the distribution and collection of completed questionnaires. Three research group staff members coordinated the survey, which was sent *via* an online platform with the necessary guidelines and instructions. Each survey was completed and submitted anonymously by GPs and managers of PHC institutions in Tibet, and the leader of the research group reviewed the quality of the collected questionnaires. Questionnaires were excluded from the database if they were incomplete or if there were any data quality issues identified during the review process.

### Statistical analysis

All calculations were conducted using the Statistical Package for Social Sciences (SPSS) for Mac (Version 26.0, SPSS, Inc., Chicago, IL, USA). Descriptive statistics (frequencies and percentages) were used to describe the characteristics of training needs. In this research, we clarified our research hypotheses before conducting multiple comparisons. Our study focused on several key indicators, mainly age, job position, and degree. For each indicator, we formulated the corresponding research hypotheses namely: (1) There are differences in CME training needs among GPs of different ages in Tibet, (2) There are differences in CME training needs among GPs of different job positions in Tibet, (3) There is a difference in the demand for CME training among GPs with different degrees in Tibet. For the first test hypothesis, we used the 2*3 chi-square test to test whether there is a difference between the entries of different ages of GPs in Tibet in terms of the need for CME and training, and for the entries where there is a difference, we continued to use the Bonferroni correction method to make a two-by-two comparison of whether there is a difference between the different age groups. In this way, we can reduce the risk of type I errors while maintaining statistical significance. For the 2nd and 3rd test hypotheses, whether there are differences in each entry of continuing education and training needs among GPs of different positions and education levels in Tibet, we analyzed them using a 2*2 chi-square test. The tests were two-sided, and a value of *p* < 0.05 was considered statistically significant.

## Results

### Demographic characteristics

A total of 854 GPs from 7 cities in the Tibet Autonomous Region participated in the questionnaire. We excluded 42 questionnaires due to incomplete items or repeated duplicate completion, and 812 valid questionnaires were finally collected, resulting in a response rate of 95.1% (812/845). We share the specific distribution of the number of valid questionnaires in eTable 1 in the supplementary material.

Table 1 shows the social demographics and characteristics of the participants. In total, 62.4% (507/812) of the participants were female, while 37.6% (305/812) were male. Overall, 62.9% (511/812) of the GPs were under the age of 30, and only 10.3% (84/812) were over 40. We found that 13.7% (111/812) of the GPs were administrative managers, while 38.8% (315/812) were neither practicing physicians nor assistant practicing physicians. Only 8.1% (66/812) of the participants had middle-level or higher titles, while the majority of participants had a bachelor’s degree or higher (68.5%, 556/812).

**Table 1:** Demographic characteristics of GPs in primary health care institutions (*n* = 812)

Items	Category	n (%)
Sex	Female	507 (62.4)
	Male	305 (37.6)
Age, years	≤ 30	511 (62.9)
	> 30, < 40	217 (26.7)
	≥ 40	84 (10.3)
Work seniority, years	≤ 5	436 (53.7)
	> 5	376 (46.3)
Managers in primary health care institutions	Yes	111 (13.7)
	No	701 (86.3)
Practicing categories	Practicing physician	354 (43.6)
	Assistant practicing physician	143 (17.6)
	Others	315 (38.8)
Professional positions	Junior-level	746 (91.9)
	Middle-level and above	66 (8.1)
Degree	Less than bachelor degree	256 (31.5)
	Bachelor degree and above	556 (68.5)

### Training needs of GP participants in Tibet

**Fig. 1:**
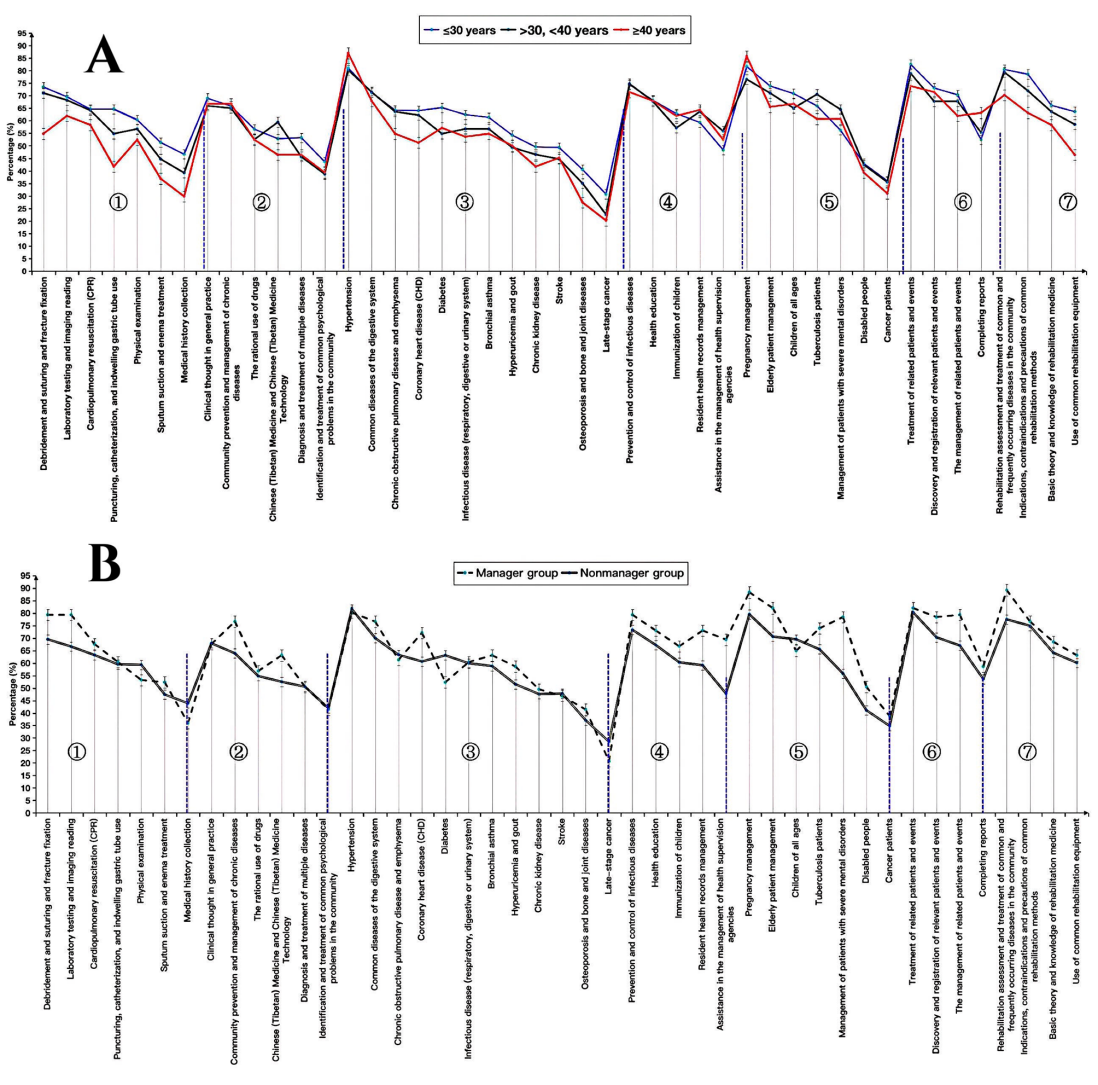
Training needs for GPs of different categories. **Fig. 1A** Training needs for GPs of different ages. “Various laboratory tests and imaging assignments” ranked first in terms of clinical skills training needs for the group over 40 years of age, but among the other categories, the main training needs of GPs were the same across different age groups. Fig. **Fig. 1B** Training needs for GPs of different job positions. “Community prevention and management of chronic diseases” ranked first in terms of training needs for community-based chronic disease management skills for the manager group, but for the other categories, the main training needs of GPs were the same among the two job position groups. Capital letters in Fig. 1A and Fig. 1B are defined as follows: (1) Clinical skills training needs. (2) Community-based chronic disease management skill training needs. (3) Common chronic disease training needs. (4) Public health service skills training needs. (5) Special population health management training needs. (6) Training needs in reporting and handling infectious diseases and public health emergencies. (7) Rehabilitation technique training needs

Table 2 describes the identified training needs of all participants. The seven main training needs of GPs relate to “debridement and suturing and fracture fixation” (70.8%, 575/812), “GP clinical diagnosis and treatment analysis” (67.9%, 551/812), “hypertension” (81.4%, 661/812), “prevention and control of infectious diseases” (74.1%, 602/812), “pregnancy management” (80.7%, 655/812), “treatment of related patients and events” (80.5%, 654/812), and “rehabilitation assessment and treatment of common and frequently occurring diseases in the community” (79.1%, 642/812). We can see the differences in the training needs of GPs by age and job position group in Fig. 1A and B.

**Table 2:** Training needs of GPs in primary health care institutions in Tibet (*n* = 812)

Category	n (%)
**Clinical skill training needs**	
Debridement and suturing and fracture fixation	575 (70.8)
Laboratory testing and imaging reading	555 (68.3)
Cardiopulmonary resuscitation	518 (63.8)
Puncturing, catheterization, and indwelling gastric tube use	484 (59.6)
Physical examination	475 (58.5)
Sputum suction and enema treatment	390 (48.0)
Medical history collection	348 (42.9)
**Community chronic disease management skill training needs**	
Clinical thought in general practice	551 (67.9)
Community prevention and management of chronic diseases	533 (65.6)
The rational use of drugs	447 (55.0)
Chinese (Tibetan) Medicine and Chinese (Tibetan) Medicine Technology	438 (53.9)
Diagnosis and treatment of multiple diseases	410 (50.5)
Identification and treatment of common psychological problems in the community	340 (41.9)
**Common chronic disease training needs**	
Hypertension	661 (81.4)
Common diseases of the digestive system	576 (70.9)
Chronic obstructive pulmonary disease and emphysema	512 (63.1)
Coronary heart disease	505 (62.2)
Diabetes	500 (61.6)
Infectious disease (respiratory, digestive or urinary system)	487 (60.0)
Bronchial asthma	482 (59.4)
Hyperuricemia and gout	426 (52.5)
Chronic kidney disease	389 (47.9)
Stroke	387 (47.7)
Osteoporosis and bone and joint diseases	306 (37.7)
Late-stage cancer	223 (27.5)
**Public health service skill training needs**	
Prevention and control of infectious diseases	602 (74.1)
Health education	552 (68.0)
Immunization of children	497 (61.2)
Resident health records management	495 (61.0)
Assistance in the management of health supervision agencies	412 (50.7)
**Special population health management training needs**	
Pregnancy management	655 (80.7)
Elderly patient management	586 (72.2)
Children of all ages	559 (68.8)
Tuberculosis patients	541 (66.6)
Management of patients with severe mental disorders	478 (58.9)
Disabled people	344 (42.4)
Cancer patients	287 (35.3)
**Training needs for infectious diseases and handling of public health emergencies**	
Treatment of related patients and events	654 (80.5)
Discovery and registration of relevant patients and events	580 (71.4)
The management of related patients and events	558 (68.7)
Completing reports	441 (54.3)
**Rehabilitation technology training needs**	
Rehabilitation assessment and treatment of common and frequently occurring diseases in the community	642 (79.1)
Indications, contraindications and precautions of common rehabilitation methods	610 (75.1)
Basic theory and knowledge of rehabilitation medicine	525 (64.7)
Use of common rehabilitation equipment	491 (60.5)

### Comparison of differences in training needs of GPs from different age groups

Table 3 shows the variation and types of training needs of GPs in different age groups. Compared to the 30–40 age group, GPs under 30 years of age noted more significant training needs in “puncturing, catheterization, and indwelling gastric tube use” (64.6% vs. 54.8%, *p* = 9.5 × 10^− 6^), “sputum suction and enema treatment” (51.3% vs. 44.7%, *p* = 0.026), “medical history collection” (46.6% vs. 39.2%, *p* = 0.007), “osteoporosis and bone and joint disease ” (40.5% vs. 35.0%, *p* = 0.045), “late-stage cancer” (30.7% vs. 22.6%, *p* = 0.023), and “indications, contraindications, and precautions of common rehabilitation methods” (78.5% vs. 71.9%, *p* = 0.005). GPs over 40 years of age prioritized diabetes training needs (57.1% vs. 54.8%, *p* = 0.022) more than GPs in the 30-40-year age group.

**Table 3:** Comparison of training needs of GPs of different ages in primary health care institutions [n(%)]

Category	≤ 30 (*n* = 511)	> 30, < 40 (*n* = 217)	≥ 40 (*n* = 84)	x^2^	*p*
**Clinical skill training needs**					
Debridement and suturing and fracture fixation	375^b^ (73.4)	154^a^ (71.0)	46^b^ (54.8)	12.110	**0.002**
Laboratory testing and imaging reading	355 (69.5)	148 (68.2)	52 (61.9)	1.912	0.384
Cardiopulmonary resuscitation	330 (64.6)	139 (64.1)	49 (58.3)	1.227	0.541
Puncturing, catheterization, and indwelling gastric tube use	330^b^ (64.6)	119^a^ (54.8)	35^b^ (41.7)	18.525	**9.5 × 10** ^**− 6**^
Physical examination	308 (60.3)	123 (56.7)	44 (52.4)	2.253	0.324
Sputum suction and enema treatment	262^b^ (51.3)	97^a^ (44.7)	31^b^ (36.9)	7.281	**0.026**
Medical history collection	238^b^ (46.6)	85^a^ (39.2)	25^b^ (29.8)	9.971	**0.007**
**Community chronic disease management skill training needs**					
Clinical thought in general practice	352 (68.9)	143 (65.9)	56 (66.7)	0.684	0.711
Community Prevention and Management of Chronic Diseases	336 (65.8)	141 (65.0)	56 (66.7)	0.084	0.959
The rational use of drugs	289 (56.6)	114 (52.5)	44 (52.4)	1.265	0.531
Chinese (Tibetan) Medicine and Chinese (Tibetan) Medicine Technology	270 (52.8)	129 (59.4)	39 (46.4)	4.806	0.090
Diagnosis and treatment of multiple diseases	272 (53.2)	99 (45.6)	39^a^ (46.4)	4.145	0.126
Identification and treatment of common psychological problems in the community	223 (43.6)	84 (38.7)	33 (39.3)	1.779	0.411
**Common chronic disease training needs**					
Hypertension	414 (81.0)	174 (80.2)	73 (86.9)	1.943	0.379
Common diseases of the digestive system	364 (71.2)	155 (71.4)	57 (67.9)	0.434	0.805
Chronic obstructive pulmonary disease and emphysema	328 (64.2)	138 (63.6)	46 (54.8)	2.789	0.248
Coronary heart disease	327 (64.0)	135 (62.2)	43 (51.2)	5.028	0.081
Diabetes	333^b^ (65.2)	119^b^ (54.8)	48^a^ (57.1)	7.645	**0.022**
Infectious disease (respiratory, digestive or urinary system)	319 (62.4)	123 (56.7)	45 (53.6)	3.695	0.158
Bronchial asthma	313 (61.3)	123 (56.7)	46 (54.8)	2.140	0.343
Hyperuricemia and gout	277 (54.2)	107 (49.3)	42 (50.0)	1.694	0.429
Chronic kidney disease	253 (49.5)	101 (46.5)	35 (41.7)	1.999	0.368
Stroke	252 (49.3)	97 (44.7)	38 (45.2)	1.521	0.468
Osteoporosis and bone and joint diseases	207^b^ (40.5)	76^a^ (35.0)	23^b^ (27.4)	6.188	**0.045**
Late-stage cancer	157^b^ (30.7)	49^a^ (22.6)	17^a^ (20.2)	7.526	**0.023**
**Public health service skill training needs**					
Prevention and control of infectious diseases	380 (74.4)	162 (74.7)	60 (71.4)	0.365	0.833
Health education	348 (68.1)	147 (67.7)	57 (67.9)	0.010	0.995
Immunization of children	321 (62.8)	124 (57.1)	52 (61.9)	2.085	0.353
Resident Health Records Management	303 (59.3)	138 (63.6)	54 (64.3)	1.618	0.445
Assistance in the management of health supervision agencies	247 (48.3)	121 (55.8)	44 (52.4)	3.460	0.177
**Special population health management training needs**					
Pregnancy management	417 (81.6)	166 (76.5)	72 (85.7)	4.079	0.130
Elderly patient management	377 (73.8)	154 (71.0)	55 (65.5)	2.687	0.261
Children of all ages	362 (70.8)	141 (65.0)	56 (66.7)	2.649	0.266
Tuberculosis patients	337 (65.9)	153 (70.5)	51 (60.7)	2.895	0.235
Management of patients with severe mental disorders	287 (56.2)	140 (64.5)	51 (60.7)	4.520	0.104
Disabled people	219 (42.9)	92 (42.4)	33 (39.3)	0.377	0.828
Cancer patients	184 (36.0)	77 (35.5)	26 (31.0)	0.809	0.667
**Training needs for infectious diseases and handling of public health emergencies**					
Treatment of related patients and events	421 (82.4)	171 (78.8)	62 (73.8)	3.959	0.138
Discovery and registration of relevant patients and events	373 (73.0)	147 (67.7)	60 (71.4)	2.059	0.357
The management of related patients and events	359 (70.3)	147 (67.7)	52 (61.9)	2.471	0.291
Completing reports	268 (52.4)	120 (55.3)	53 (63.1)	3.414	0.181
**Rehabilitation technology training needs**					
Rehabilitation assessment and treatment of common and frequently occurring diseases in the community	411 (80.4)	172 (79.3)	59 (70.2)	4.535	0.104
Indications, contraindications and precautions of common rehabilitation methods	401^b^ (78.5)	156^a^ (71.9)	53^b^ (63.1)	10.786	**0.005**
Basic theory and knowledge of rehabilitation medicine	338 (66.1)	138 (63.6)	49 (58.3)	2.072	0.355
Use of common rehabilitation equipment	325^b^ (63.6)	127^a^ (58.5)	39^b^ (46.4)	9.367	**0.009**

Note: In this study, the results of pairwise comparisons were marked by superscript (^a, b^). If the marker letters were the same between the two groups, the difference between the two groups was not statistically significant. If the two groups of marker letters are different, the difference between these two groups is statistically significant. The black fonts in the table indicate statistically significant differences

### Comparison of differences in training needs of GPs of different job positions and education backgrounds

Table 4 shows that when compared to nonmanager GPs, manager GPs have more training needs in “debridement and suturing and fracture fixation” (79.3% vs. 69.5%, *p =* 0.035), “laboratory testing and imaging reading” (79.3% vs. 66.6%, *p =* 0.008), “community prevention and management of chronic diseases” (76.6% vs. 63.9%, *p* = 0.009), “Chinese (Tibetan) Medicine and Chinese (Tibetan) Medicine Technology” (63.1% vs. 52.5%, *p =* 0.038), “coronary heart disease (CHD) ” (72.1% vs. 60.6%, *p =* 0.021), “resident health records management” (73.0% vs. 59.1%, *p =* 0.005), “assistance in the management of health supervision agencies” (69.4% vs. 47.8%, *p =* 2.4 × 10^− 6^), “pregnancy management” (88.3% vs. 79.5%, *p =* 0.029), “elderly patient management” (82.0% vs. 70.6%, *p =* 0.013), “treatment of patients with severe mental disorders” (78.4% vs. 55.8%, *p =* 7.0 × 10^− 6^), and “the management of related patients and events” (79.3% vs. 67.0%, *p =* 0.010) and fewer training needs in “diabetes ” (52.3% vs. 61.3%, *p =* 0.030).

**Table 4:** Training needs of GPs with different job positions and degrees in primary health care institutions [n(%)]

Category	Manager group (*n* = 111)	Nonmanager group (*n* = 701)	x^2^	*p*	Less than bachelor degree (*n* = 256)	Bachelor degree and above (*n* = 556)	x^2^	*p*
**Clinical skill training needs**								
Debridement and suturing and fracture fixation	88 (79.3)	487 (69.5)	4.459	**0.035**	168 (65.6)	407 (73.2)	4.868	**0.027**
Laboratory testing and imaging reading	88 (79.3)	467 (66.6)	7.100	**0.008**	149 (58.2)	406 (73.0)	17.793	**2.5 × 10** ^**− 6**^
Cardiopulmonary resuscitation	75 (67.6)	443 (63.2)	0.793	0.373	158 (61.7)	360 (64.7)	0.697	0.404
Puncturing, catheterization, and indwelling gastric tube use	67 (60.4)	417 (59.5)	0.030	0.862	130 (50.8)	354 (63.7)	12.092	**0.001**
Physical examination	59 (53.2)	416 (59.3)	1.513	0.219	150 (58.6)	325 (58.5)	0.001	0.970
Sputum suction and enema treatment	58 (52.3)	332 (47.4)	0.918	0.338	104 (40.6)	286 (51.4)	8.212	**0.004**
Medical history collection	40 (36.0)	308(43.9)	2.443	0.118	108 (42.2)	240 (43.2)	0.068	0.794
**Community chronic disease management skill training needs**								
Clinical thought in general practice	75 (67.6)	476 (67.9)	0.005	0.944	177 (69.1)	374 (67.3)	0.282	0.595
Community prevention and management of chronic diseases	85 (76.6)	448 (63.9)	6.818	**0.009**	161 (62.9)	372 (66.9)	1.253	0.263
The rational use of drugs	63 (56.8)	384 (54.8)	0.151	0.697	126 (49.2)	321 (57.7)	5.136	**0.023**
Chinese (tibetan) medicine and chinese (tibetan) medicine technology	70 (63.1)	368 (52.5)	4.306	**0.038**	115 (44.9)	323 (58.1)	12.241	**4.7 × 10** ^**− 6**^
Diagnosis and treatment of multiple diseases	56 (50.5)	354 (50.5)	9.1 × 10^− 6^	0.992	123 (48.0)	287 (51.6)	0.895	0.344
Identification and treatment of common psychological problems in the community	46 (41.4)	294 (41.9)	0.010	0.921	104 (40.6)	236 (42.4)	0.239	0.625
**Common chronic disease training needs**								
Hypertension	89 (80.2)	572 (81.6)	0.127	0.721	203 (79.3)	458 (82.4)	1.097	0.295
Common diseases of the digestive system	85 (76.6)	491 (70.0)	1.984	0.159	168 (65.6)	408 (73.4)	5.115	**0.024**
Chronic obstructive pulmonary disease and emphysema	68 (61.3)	444 (63.3)	0.177	0.674	146 (57.0)	366 (65.8)	5.822	**0.016**
Coronary heart disease	80 (72.1)	425 (60.6)	5.338	**0.021**	144 (56.3)	361 (64.9)	5.614	**0.018**
Diabetes	58 (52.3)	442 (63.1)	4.725	**0.030**	162 (63.3)	338 (60.8)	0.459	0.498
Infectious disease (respiratory, digestive or urinary system)	67 (60.4)	420 (59.9)	0.008	0.929	147 (57.4)	340 (61.2)	1.016	0.314
Bronchial asthma	70 (63.1)	412 (58.8)	0.731	0.393	142 (55.5)	340 (61.2)	2.346	0.126
Hyperuricemia and gout	65 (58.6)	361 (51.5)	1.916	0.166	112 (43.8)	314 (56.5)	11.381	**0.001**
Chronic kidney disease	55 (49.5)	334 (47.6)	0.139	0.709	103 (40.2)	286 (51.4)	8.818	**0.003**
Stroke	52 (46.8)	335 (47.8)	0.034	0.854	103 (40.2)	284 (51.1)	8.264	**0.004**
Osteoporosis and bone and joint diseases	46 (41.4)	260 (37.1)	0.773	0.379	81 (31.6)	225 (40.5)	5.816	**0.016**
Late-stage cancer	23 (20.7)	200 (28.5)	2.934	0.087	65 (25.4)	158 (28.4)	0.806	0.369
**Public health service skill training needs**								
Prevention and control of infectious diseases	88 (79.3)	514 (73.3)	1.773	0.183	183 (71.5)	419 (75.4)	1.373	0.241
Health education	81 (73.0)	471 (67.2)	1.472	0.225	173 (67.6)	379 (68.2)	0.028	0.868
Immunization of children	74 (66.7)	423 (60.3)	1.614	0.204	151 (59.0)	346 (62.2)	0.778	0.378
Resident health records management	81 (73.0)	414 (59.1)	7.796	**0.005**	153 (59.8)	342 (61.5)	0.224	0.636
Assistance in the management of health supervision agencies	77 (69.4)	335 (47.8)	17.855	**2.4 × 10** ^**− 6**^	141 (55.1)	271 (48.7)	2.816	0.093
**Training needs for infectious diseases and handling of public health emergencies**								
Pregnancy management	98 (88.3)	557 (79.5)	4.791	**0.029**	198 (77.3)	457 (82.2)	2.644	0.104
elderly patient management	91 (82.0)	495 (70.6)	6.166	**0.013**	170 (66.4)	416 (74.8)	6.178	**0.013**
Children of all ages	72 (64.9)	487 (69.5)	0.948	0.330	174 (68.0)	385 (69.2)	0.133	0.715
Tuberculosis patients	82 (73.9)	459 (65.5)	3.038	0.081	170 (66.4)	371 (66.7)	0.008	0.928
Management of patients with severe mental disorders	87 (78.4)	391 (55.8)	20.215	**7.0 × 10** ^**− 6**^	155 (60.5)	323 (58.1)	0.436	0.509
Disabled people	56 (50.5)	288 (41.1)	3.443	0.064	104 (40.6)	240 (43.2)	0.463	0.496
Cancer patients	43 (38.7)	244 (34.8)	0.648	0.421	88 (34.4)	199 (35.8)	0.154	0.695
**Training needs for reporting and handling infectious diseases and public health emergencies**								
Treatment of related patients and events	91 (82.0)	563 (80.3)	0.170	0.680	204 (79.7)	450 (80.9)	0.174	0.676
Discovery and registration of relevant patients and events	87 (78.4)	493 (70.3)	3.043	0.081	186 (72.7)	394 (70.9)	0.276	0.599
The management of related patients and events	88 (79.3)	470 (67.0)	6.670	**0.010**	160 (62.5)	398 (71.6)	6.727	**0.009**
Completing reports	65 (58.6)	376 (53.6)	0.935	0.334	143 (55.9)	298 (53.6)	0.362	0.548
**Rehabilitation technology training needs**								
Rehabilitation assessment and treatment of common and frequently occurring diseases in the community	99 (89.2)	543 (77.5)	7.963	**0.005**	188 (73.4)	454 (81.7)	7.150	**0.007**
Indications, contraindications and precautions of common rehabilitation methods	85 (76.6)	525 (74.9)	0.145	0.703	170 (66.4)	440 (79.1)	15.201	**9.7 × 10** ^**− 6**^
Basic theory and knowledge of rehabilitation medicine	76 (68.5)	449 (64.1)	0.818	0.366	162 (63.3)	363 (65.3)	0.309	0.578
Use of common rehabilitation equipment	70 (63.1)	421 (60.1)	0.362	0.547	136 (53.1)	355 (63.8)	8.433	**0.004**

Notes: Black fonts in the table indicate statistically significant differences

Furthermore, GPs with a bachelor’s degree or higher have more training needs than those with less than a bachelor’s degree in “debridement and suturing and fracture fixation” (73.2% vs. 65.6%, *p =* 0.027), “various laboratory tests and imaging readings” (73.0% vs. 58.2%, *p =* 2.5 × 10^− 6^), “puncturing, catheterization, and indwelling gastric tube use” (63.7% vs. 50.8%, *p =* 0.001), “sputum suction and enema treatment” (51.4% vs. 40.6%, *p =* 0.004), “the rational use of drugs” (57.7% vs. 49.2%, *p =* 0.023), “Chinese (Tibetan) Medicine and Chinese (Tibetan) Medicine Technology” (58.1% vs. 44.9%, *p =* 4.7 × 10^− 6^), “common diseases of the digestive system” (73.4% vs. 65.6%, *p =* 0.024), “chronic obstructive pulmonary disease and emphysema” (65.8% vs. 57.0%, *p =* 0.016), “CHD” (64.9% vs. 56.3%, *p =* 0.018), “hyperuricaemia and gout” (56.5% vs. 43.8%, *p =* 0.001), “chronic kidney disease” (51.4% vs. 40.2%, *p =* 0.003), “stroke” (51.1% vs. 40.2%, *p =* 0.001), “osteoporosis and bone and joint diseases” (40.5% vs. 31.6%, *p =* 0.016), “elderly patient management” (74.8% vs. 66.4%, *p =* 0.013), and “management of related patients and events” (71.6% vs. 62.5%, *p =* 0.009).

## Discussion

### The main findings

Our study investigated the training needs of GPs in the Tibetan region, a unique plateau area characterized by slower development of general practice and lower medical skill levels among GPs compared to mainland China. Our findings revealed a significant demand for professional development training among Tibetan GPs, particularly in the areas of hypertension, pregnancy management, related patient events, trauma and fracture management, emergency medical care, maternity care, and traditional Chinese medicine (TCM) techniques. The disparity in training needs was noted across different age groups, job positions, and educational backgrounds, which is indicative of the varied professional development requirements in this region.

Comparing our findings with existing literature, we found consistency in the demand for training in hypertension and pregnancy management, highlighting the significance of addressing these specific needs [7, 18]. The high prevalence of hypertension in Tibet, potentially linked to environmental factors such as low oxygen levels and dietary habits, underscores the critical need for tailored training in this area [19]. Additionally, the scarcity of obstetricians and gynecologists in Tibet, coupled with suboptimal obstetric and gynecological care, contributes to a higher maternal morbidity rate, aligning with our identification of pregnancy management as a key training need [20]. The elevated prevalence of chronic diseases in Tibet, influenced by its unique geographic environment and dietary patterns, emphasizes the necessity of targeted medical training in these specific domains [21, 22]. Consequently, our study emphasizes the urgent need for tailored training programs to address the specific healthcare challenges in the Tibetan region.

### GP training in trauma and fracture management, emergency medical care, and maternity care is essential

It is worth noting that the CME content needs of the participating GPs varied, and the precision of CME material should be enhanced to improve its applicability [23]. Simple fracture and trauma management is a basic community first aid skill. According to the findings of this study, the leading training needs in terms of GPs’ clinical skills in Tibet relate to debridement, suturing, and fracture fixation. However, in a prior study of GP training needs by West China Hospital of Sichuan University, this skill placed 5th out of 23 items (5/23) [7]. Because Tibet is located on a plateau, transportation is challenging, there are many herdsmen, and traffic accidents and accidental injuries occur more frequently in the local population [24]. According to a study conducted in the United States, there are very few emergency doctors in rural areas, and GPs must perform the majority of emergency treatment procedures, thus necessitating the extension of GP training in rural areas [25]. This is consistent with the findings of the current study, which found strong demand for training in emergency and first aid capabilities among GPs in rural Tibetan areas. This may be due to a lack of emergency physicians and an inadequate level of emergency care and treatment techniques and capabilities among PHC GPs.

Notably, one retrospective analysis of maternal mortality in 34 Chinese provinces and cities from 1990 to 2017 revealed that while Tibet still had the highest maternal mortality rate nationally in the period (82.7 per 100,000 live births), it had fallen by 74.4% since 1990 [26]. Although the maternal mortality rate in Tibet has dropped in recent years as a result of rapid economic development and the widespread implementation of health care policy, there is still a substantial disparity relative to other Chinese provinces [27]. This could be the main cause of the strong demand for maternal special population training among GPs in Tibetan areas, as revealed by our findings. Therefore, it is imperative to improve the maternal management capacity and capabilities of local PHC GPs in Tibet.

### The training needs of different categories of GPs have different focuses

Our findings show that junior GPs (< 40 years of age) had more need for training in “debridement and suturing and fracture fixation”, “puncturing, catheterization, and indwelling gastric tube use”, “sputum suction and enema treatment”, “medical history collection” and rehabilitation-related knowledge” than senior GPs (≥ 40 years of age). There may be several reasons for this result. First, many junior GPs have only recently started working and have only undergone short-term training. They often lack relevant theoretical knowledge, clinical experience, and skills. Second, the majority of junior GPs have more motivation to learn [28, 29]. Of course, senior GPs do not have extra time to attend training due to their work and family responsibilities, which may be a major reason for their fewer training needs.

Most PHC institution managers have transferred from other specialties, lacking systematic study and training in general practice and essential management expertise, particularly in Tibet [30]. Our findings reveal that managers of PHC institutions emphasize “community prevention and management of chronic diseases”, “resident health records administration”, and “assistance in the management of health supervision agencies” more than nonmanagers, while nonmanagers prioritize diabetes training. Although the Tibetan region has numerous medical specialists from China’s developed regions for long-term support, when experts leave Tibet’s local medical level and capacity frequently fail to meet local needs [31]. This could be why local PHC managers have greater training needs in the above areas. Management of special populations (mothers, the elderly, patients with severe mental problems or chronic conditions, etc.) is a significant responsibility of GPs and an important indicator of the quality of medical services offered by medical institutions [32]. According to our findings, there is a greater need for training for PHC institution managers in the management of special populations. As a result, more management training for Tibetan PHC institution managers, as well as comprehensive clinical training for nonmanager GPs, are advocated as a significant means to address out-of-date and missing skills among local primary medical-level staff.

TCM plays a unique role in prevention, health care, wellness, and rehabilitation, and it is widely used in Chinese PHC institutions. Tibetan medicine has facilitated the social development of numerous ethnic groups on the Tibetan Plateau and in the Himalayan region [33]. Our study found that those with a bachelor’s degree or higher had more training demands in TCM (Tibetan) medicine techniques than those with a lower degree. This disparity could be attributed to differences in learning aptitudes and interests among GPs of various educational backgrounds and to the significance of their understanding of regional ethnic medicine practice. Tibetan medicine is very popular among Tibetan residents [34]. Therefore, we should strengthen Tibetan medicine knowledge training and learning for local GPs and guide the continuity of practice among local GPs in their treatment work through the establishment of corresponding treatment protocols and guidelines.

### Limitations

Our study has several limitations. Firstly, face-to-face interviews were difficult due to the Tibetan Plateau’s hypoxic natural environment, the remoteness of its rural areas, inconvenient local transportation, and language difficulties among Tibetans. Secondly, we conducted an online survey and were unable to identify some subtle differences that can be gained from the field, which may affect the answers obtained and the understanding of certain research questions. Thirdly, we did not compare the training needs of GPs in rural and urban areas of Tibet. In addition, the participants in this study could not fully represent all local GPs in Tibet. This is because we could not fully control the age and gender ratio of the participants during the actual research process. Due to a variety of factors, such as willingness to participate and availability, we could only strive to make the characteristics of the participants as close as possible to the overall characteristics of GPs in Tibet. Besides, another limitation of our study is the lack of detailed quantitative analysis of the needs across different dimensions, which may limit a comprehensive understanding of the degree of needs among general practitioners. In future research, we plan to introduce a more refined scoring system to supplement and deepen our understanding in this area. Finally, our questionnaire was primarily a subjective self-report survey, and our lack of objective survey evaluation may have affected the study outcomes. More residents will participate as researchers in the survey to provide guidance and incorporate more objective scales to improve the comprehensiveness of research to address the aforementioned limitations and issues in future research.

## Conclusion

In conclusion, our study emphasizes the urgent need for tailored training programs to address the specific healthcare challenges in the Tibetan region. By focusing on hypertension, pregnancy management, and trauma care, we can better equip GPs to provide comprehensive care. These findings underscore the importance of personalized training initiatives that consider the unique sociodemographic characteristics and healthcare landscape of Tibet. Meanwhile, we call for graded, stratified, and targeted GP training to meet the needs of GPs in Tibetan areas, the cultivation of specialized and versatile GPs, and addressing insufficient professional knowledge among GPs and underdeveloped PHC in many Tibetan areas. Ultimately, addressing these training needs has the potential to significantly improve the quality of primary healthcare services in the region.

### Electronic supplementary material

Below is the link to the electronic supplementary material.

Supplementary Material 1

Supplementary Material 2

## Data Availability

The datasets generated during and/or analyzed during the current study are available from the corresponding author on reasonable request.

## Abbreviations

GP: General Practitioner
CME: Continuing Medical Education
PHC: Primary Health Care
WONCA: World Organization of Family Doctors
QR: Quick Response
SPSS: Statistical Package for Social Sciences
CHD: Coronary Heart Disease
TCM: Traditional Chinese Medicine
