# Supplementary material for: Current status and continuing medical education need for general practitioners in Tibet, China: a cross-sectional study
Source: BMC Med Educ. 2024 Mar 8;24:265. doi: 10.1186/s12909-024-05143-5 (PMC10924353; doi:10.1186/s12909-024-05143-5)
Supplement: Supplementary file 1 — Supplementary Material 1 [file 12909_2024_5143_MOESM1_ESM.docx]

**eTable 1** General practitioners from 7 cities in the Tibet autonomous region (n = 812)

| Districts | Lhasa  City | Xigaze City | Nyingchi City | Qamdo City | Shannan  City | Nagqu City | Ngari Prefecture | Total |
| --- | --- | --- | --- | --- | --- | --- | --- | --- |
| No. of GPs from involved primary healthcare institutions | 435 | 150 | 19 | 41 | 75 | 68 | 24 | 812 |
