# Supplementary material for: Current status and continuing medical education need for general practitioners in Tibet, China: a cross-sectional study
Source: BMC Med Educ. 2024 Mar 8;24:265. doi: 10.1186/s12909-024-05143-5 (PMC10924353; doi:10.1186/s12909-024-05143-5)
Supplement: Supplementary file 2 — Supplementary Material 2 [file 12909_2024_5143_MOESM2_ESM.doc]

**Additional file 1**

**A questionnaire investigation of training needs of general practitioners working in primary health care institutions in Tibet**

Number ______________

City/District/State _______________

District _______________

Primary health care institution ______________

Investigate date: ____________ Signature of investigator: ______________

Check date: _____________ Signature of scrutator: _______________

**Dear doctors:**

This study was conducted to improve the disease management of general practitioners in primary health care institutions in Tibetan areas. The questionnaire will only be used to investigate your training needs. Your thoughts are important to improving the training program. Please take a few minutes to answer the following questions carefully. We can assure you that your answers will be kept strictly confidential.

Thank you for your support.

General Practice Research Team of Yangpu Hospital affiliated with Tongji University

Note:

1.By completing this questionnaire, you are giving your informed consent to participate in this study.

2.General practitioners working in primary health care are included in the study.

3. Rehired retirees and temporary employees are excluded from the study.

(Please mark the “√” under the numbers, e.g., ① and ②, or write your answer on the “_____” following each question)

**Part 1. Social demographic characteristics**

1. Name: ________ Telephone number: ____________

2.Gender: ① Male ② Female

3.Age: ________

4.Years worked as a GP: ____________

5.Administrative positions: ________ Work areas: ________

6. What type of practicing qualification have you acquired?

① Practicing physician

② Practicing assistant physician

③ None (Please answer question 7)

7. What is the highest title level you have acquired?

① Senior title (assistant chief physician or chief physician)

② Middle title ([attending](http://cn.bing.com/dict/search?q=Attending&FORM=BDVSP6&mkt=zh-cn) [physician](http://cn.bing.com/dict/search?q=physician&FORM=BDVSP6&mkt=zh-cn))

③ Junior title and below ([resident](http://cn.bing.com/dict/search?q=resident&FORM=BDVSP6&mkt=zh-cn) [physician](http://cn.bing.com/dict/search?q=physician&FORM=BDVSP6&mkt=zh-cn) or assistant doctor)

④ None

8.Education level

① Undergraduate and above

② Junior college

③ Technical secondary school/High school

④ Below high school

**Part 2. Training needs**

9. Which of the following clinical skills do you need more training in? (Select all that apply.)

① Debridement and suturing and fracture fixation

② Laboratory testing and imaging reading (ECG, imaging, blood gas analysis, etc.)

③ Cardiopulmonary resuscitation (CPR)

④ Puncturing, catheterization, and indwelling gastric tube use

⑤ Physical examination

⑥ Sputum suction and enema treatment

⑦ Medical history collection

⑧ Other______________________

10. Which of the following community chronic disease management skills do you need more training in? (Select all that apply.)

① Community prevention and management of important chronic diseases (e.g., hypertension, diabetes, etc.)

② Clinical thought in general practice

③ Reasonable use of medication

④ Technical aspects of Chinese (Tibetan) medicine (TCM)

⑤ Multimorbidity diagnosis and treatment

⑥ Identification and management of common psychological problems in

the community

⑦ Other ________________________

11.Which of the following chronic diseases do you need more training in? Examples include community-based chronic disease intake procedures, history taking, SOAP (Subjective, Objective, Assessment, Plan) record writing, follow-up consultations, and health management service specifications. (Select all that apply).

① Hypertension

② Digestive system diseases

③ Chronic obstructive pulmonary disease and emphysema

④ Coronary heart disease (CHD)

⑤ Diabetes

⑥ Infectious diseases (respiratory, gastrointestinal or urinary system)

⑦ Bronchial asthma

⑧ Hyperuricaemia and gout

⑨ Chronic kidney disease

⑩ Stroke

⑪ Osteoporosis and Bone and Joint Diseases

⑫ Late-stage cancer

⑬ Other___­­­­­________________

12. Which of the following public health service skills do you need more training in? (Select all that apply.)

① Prevention and control of infectious diseases

② Health education

③ Planned immunization

④ Resident Health Records Management

⑤ Assistance in the management of health supervision agencies

⑥ Other___­­­­­________________

13．For which of the following populations do you have a need for training in health management? (Select all that apply.)

① Pregnancy management

② Elderly patient management

③ Children of all ages

④ Tuberculosis patients

⑤ Management of patients with severe mental disorders

⑥ Disabled people

⑦ Cancer patients

⑧ Other___­­­­­________________

14. What are your training needs in the area of communicable diseases and the handling of public health emergencies? (Select all that apply.)

① Treatment of related patients and events

② Identification and registration of relevant patients and events

③ The management of related patients and events

④ Completing reports

⑤ Other__________________

15.What are your training needs in terms of rehabilitation techniques? (Select all that apply.)

① Rehabilitation assessment and treatment of common and frequently occurring diseases in the community

② Indications, contraindications and precautions of common rehabilitation methods

③ Basic theory and knowledge of rehabilitation medicine

④ Use of common rehabilitation equipment

⑤ Other__________________

Thank you very much for taking the time to complete this survey. Your feedback is valued and very much appreciated.
